# Co-circulation of Chikungunya Virus during the 2015–2017 Zika Virus Outbreak in Pernambuco, Brazil: An Analysis of the Microcephaly Epidemic Research Group Pregnancy Cohort

**DOI:** 10.4269/ajtmh.21-0449

**Published:** 2022-04-11

**Authors:** Ludmila Lobkowicz, Demócrito de Barros Miranda-Filho, Ulisses Ramos Montarroyos, Celina Maria Turchi Martelli, Thalia Velho Barreto de Araújo, Wayner Vieira De Souza, Luciana Caroline Albuquerque Bezerra, Rafael Dhalia, Ernesto T. A. Marques, Nuria Sanchez Clemente, Jayne Webster, Aisling Vaughan, Emily L. Webb, Elizabeth B. Brickley, Ricardo Arraes de Alencar Ximenes, on behalf of the Microcephaly Epidemic Research Group

**Affiliations:** ^1^ Health Equity Action Lab, Department of Infectious Disease Epidemiology, London School of Hygiene & Tropical Medicine, London, United Kingdom;; ^2^ Departamento de Medicina Interna, Universidade de Pernambuco, Recife, Brasil;; ^3^ Instituto de Ciências Biológicas, Universidade de Pernambuco, Recife, Brasil;; ^4^ Instituto Aggeu Magalhães, Fundação Oswaldo Cruz, Recife, Brasil;; ^5^ Departamento de Medicina Social, Universidade Federal de Pernambuco, Recife, Brasil;; ^6^ Secretaria Estadual de Saúde, Pernambuco, Brasil;; ^7^ Department of Infectious Diseases and Microbiology, University of Pittsburgh, Pittsburgh, Pennsylvania;; ^8^ Department of Disease Control, London School of Hygiene & Tropical Medicine, London, United Kingdom;; ^9^ MRC International Statistics and Epidemiology Group, London School of Hygiene & Tropical Medicine, London, United Kingdom;; ^10^ Departamento de Medicina Tropical, Universidade Federal de Pernambuco, Recife, Brasil

## Abstract

Co-circulation of arthropod-borne viruses, particularly those with shared mosquito vectors like Zika (ZIKV) and Chikungunya (CHIKV), is increasingly reported. An accurate differential diagnosis between ZIKV and CHIKV is of high clinical importance, especially in the context of pregnancy, but remains challenging due to limitations in the availability of specialized laboratory testing facilities. Using data collected from the prospective pregnancy cohort study of the Microcephaly Epidemic Research Group, which followed up pregnant persons with rash during the peak and decline of the 2015–2017 ZIKV epidemic in Recife, Pernambuco, Brazil, this study aims to describe the geographic and temporal distribution of ZIKV and CHIKV infections and to investigate the extent to which ZIKV and CHIKV infections may be clinically differentiable. Between December 2015 and June 2017, we observed evidence of co-circulation with laboratory confirmation of 213 ZIKV mono-infections, 55 CHIKV mono-infections, and 58 sequential ZIKV/CHIKV infections (i.e., cases with evidence of acute ZIKV infection with concomitant serological evidence of recent CHIKV infection). In logistic regressions with adjustment for maternal age, ZIKV mono-infected cases had lower odds than CHIKV mono-infected cases of presenting with arthralgia (aOR, 99% CI: 0.33, 0.15–0.74), arthritis (0.35, 0.14–0.85), fatigue (0.40, 0.17–0.96), and headache (0.44, 0.19–1.00). However, sequential ZIKV/CHIKV infections complicated discrimination, as they did not significantly differ in clinical presentation from CHIKV mono-infections. These findings suggest clinical symptoms alone may be insufficient for differentiating between ZIKV and CHIKV infections during pregnancy and therefore laboratory diagnostics continue to be a valuable tool for tailoring care in the event of arboviral co-circulation.

## INTRODUCTION

Arthropod-borne viruses (arboviruses) are a growing global health threat and important infectious cause of disability and mortality.^1^^–^^3^ Worldwide, there are increasing reports of geographic and temporal co-circulation of arboviruses, particularly those with shared mosquito vectors.^4^^–^^6^ Growing deforestation and urbanization has led to increased vector–host interactions, while enhanced population movements and changing climates facilitate the spread of viruses and arthropod vectors to previously unaffected locations.^7^^,^^8^ The persistence of poor sanitation conditions, such as surface water storage and inadequate sewage disposal, facilitate mosquito proliferation, which may be further intensified by rising rates of insecticide resistance.^8^^,^^9^ In Latin America and the Caribbean especially, the co-circulating arboviruses Chikungunya virus (CHIKV), Dengue virus (DENV), and Zika virus (ZIKV) are of significant public health concern.^10^ The three arboviruses are primarily transmitted by *Aedes aegypti*, an urban and peri-urban mosquito, which is present in almost all tropical and subtropical areas.^3^^,^^11^ Currently over three billion people live in regions where *Ae. aegypti* is present and, as a result, live at risk of arboviral infection.^11^

A major concern regarding the co-circulation of CHIKV, DENV, and ZIKV is the accurate discrimination between types of arboviral infections, as the clinical presentations of the three arboviruses share notable similarities.^12^ For all three arboviruses, a substantial percentage of infections can occur asymptomatically: CHIKV has been described to be asymptomatic in 3–75% of infections, whereas DENV and ZIKV infections are reported to be asymptomatic in 60–75% of infections.^13^^–^^16^ Moreover, mild symptomatic cases for each of the three arboviruses present with overlapping signs and symptoms, including rash, fever, myalgia, arthralgia, conjunctivitis, and headache, although the relative frequencies of signs and symptoms for each of the arboviruses remain poorly defined.^17^^–^^20^ Additionally, laboratory diagnosis can be challenging in low-resource settings in which nucleic acid amplification and serological testing facilities are not routinely available.

Nevertheless, an accurate differential diagnosis is of high clinical importance as serious complications can manifest differently from CHIKV, DENV, and ZIKV infections. Chikungunya virus infections have been associated with neurological complications as well as persistent, disabling severe arthralgia, while DENV infection may progress to severe dengue or death.^17^^,^^18^ ZIKV infection has mainly been associated with the development of neurological complications, such as Guillain-Barré syndrome, and infections during pregnancy are of particular concern as congenital ZIKV infections can cause a range of adverse birth and neurodevelopmental outcomes, known as Congenital Zika Syndrome.^19^^,^^21^

Using data collected from a cohort of pregnant individuals with rash who were notified to the Pernambuco State Health Department surveillance system (Center for Strategic Information on Health Surveillance in Pernambuco; Cievs/PE) during the outbreak and decline of the 2015–2017 ZIKV epidemic in Recife, Pernambuco, Brazil, this study aimed to investigate co-circulating arboviruses in the context of pregnancy.^22^ The primary objectives were: 1) to characterize the geographic and temporal co-circulation of CHIKV and ZIKV and 2) to compare the clinical presentations between maternal CHIKV mono-infections, ZIKV mono-infections, and sequential ZIKV/CHIKV infections (i.e., acute ZIKV infections with concomitant evidence of recent CHIKV infection) among pregnant persons with rash. Due to the inherent obstacle of immunological cross-reactivity among flaviviruses, co-circulating DENV infections were not assessed in the current analyses.

## MATERIALS AND METHODS

### Disclosure.

The study was approved by the ethics committees of Fiocruz Pernambuco (Instituto Aggeu Magalhães, Recife, Pernambuco, Brazil; 53240816.4.0000.5190) and the London School of Hygiene & Tropical Medicine (London, United Kingdom; 16412).

### Study design.

This analysis used sociodemographic, laboratory, and clinical data from the Microcephaly Epidemic Research Group (MERG) Pregnancy Cohort (for full study protocol, see: http://scf.cpqam.fiocruz.br/merg/).^22^ The prospective cohort recruited and followed-up pregnant individuals in Pernambuco State who were notified to Cievs/PE with rash (i.e., a common symptom of acute arbovirus infection) between December 2015 and June 2017; no exclusion criteria were applied. Detailed information on the design of the cohort study, participants, and laboratory procedures has previously been reported by Ximenes and others (2019).^22^

### Laboratory testing.

The diagnostic testing of serum samples, which were collected over up to three study visits during and after pregnancy, was conducted at the Laboratorio de Virologia e Terapia Experimental of the Fundação Oswaldo Cruz (LAVITE-Fiocruz, Recife, Pernambuco, Brazil).^22^

Sera were tested for ZIKV infections using a combination of: quantitative reverse transcription polymerase chain reaction tests (qRT-PCR) using primers and probes described by Lanciotti and colleagues, U.S. Centers for Disease Control and Prevention (US CDC) capture-immunoglobulin (Ig) M enzyme-linked immunosorbent assays (ELISAs), IgG3 ELISAs, and Plaque Reduction Neutralization Tests (PRNT_50_).^22^^–^^24^ The nucleic acid amplification and serological test results were considered in relation to the timing of rash onset during pregnancy and evaluated using an evidence-graded diagnostic algorithm, as previously described.^22^ In the current analysis, participants were considered to have a confirmed ZIKV infection if they had robust, moderate, or limited evidence of ZIKV infection during pregnancy according to the MERG algorithm (Supplemental Materials).^22^ Sera were additionally tested for recent CHIKV and DENV infections using the US CDC capture-IgM ELISA.^24^ Sequential ZIKV/CHIKV infections were defined as participants with a confirmed ZIKV infection, as described above, and a simultaneously positive CHIKV IgM test (i.e., indicating recent CHIKV infection, within 3–4 days and up to 2 months after ZIKV infection, as defined by the WHO).^25^ To reduce the potential for misclassification arising from immunological cross-reactivity among flaviviruses, participants testing positive for DENV IgM were excluded from the statistical analyses comparing the clinical presentations of ZIKV and sequential ZIKV/CHIKV infections versus CHIKV infections. Sera were also tested for recent infections with selected TORCH agents (i.e., *Toxoplasma gondii*, Parvovirus B19, Cytomegalovirus, Herpes simplex virus) by IgM ELISA.^26^

### Data analysis.

To investigate the temporal distribution of CHIKV and ZIKV infections in the cohort between December 2015 and June 2017, an epidemiological curve was plotted, presenting the number pregnant individuals with the evidence of CHIKV and ZIKV infections who were enrolled in the study from the Cievs/PE surveillance system by week of reported rash onset.

To visualize the geographical co-circulation of CHIKV and ZIKV within the cohort, ArcGIS software (ArcGIS, release 10.5. Redlands, CA) was used to geo-reference the residence of the subset of CHIKV and ZIKV-positive participants who resided in Recife and had available residence data by plotting a layer for ZIKV infections, overlayed with one for CHIKV infections onto a cartographic shapefile downloaded from the Brazilian Institute of Geography and Statistics (*Instituto Brasileiro de Geografia e Estatistica,* IBGE) website (https://mapas.ibge.gov.br/bases-e-referenciais/bases-cartograficas/malhas-digitais.html).^27^^,^^28^ The map was made at a scale of 1:100,000, which produces an error of approximately 20 m on the real scale; therefore, the residence of each infected participant is represented as a broad circle of approximately 1,250 m^2^ within the highly urbanized city. For CHIKV and ZIKV infections, Moran’s I index was calculated separately to assess spatial autocorrelations of the respective infections based on residence; sequential ZIKV/CHIKV infections were included in both calculations.^29^

To compare the clinical presentations, logistic regression models were used to assess the associations between specific signs and symptoms and infection status. Models were adjusted for maternal age as a continuous variable to account for confounding due to potential age-related differences in immunological experience and symptom presentations. To adjust for multiple testing, 99% confidence intervals were presented and results with *P* value < 0.01 were interpreted as significant. To determine whether a combination of signs and symptoms could be more predictive of being CHIKV mono-infected versus ZIKV mono-infected than individual signs and symptoms, logistic regression was used to conduct predictive modeling. Likelihood ratio tests were used to assess the goodness of fit of models containing multiple signs and symptoms versus models with only one sign or symptom (e.g., model with joint pain + joint swelling versus model with joint pain alone). Pairwise correlations in the reporting of specific signs and symptoms were investigated using χ^2^ tests.

## RESULTS

### Study population.

Of the 707 pregnant persons with rash notified to the Pernambuco State Health Department between December 2015 and June 2017, 694 (98%) were recruited for further follow-up in the MERG Pregnancy Cohort. The majority (79%) resided in the Recife city area (Table 1). The median age of participants was 25.5 years (Interquartile range [IQR]: 21, 31). The study sample self-identified most frequently as Mixed-race Brazilian (*parda*) (65%), White-Brazilian (*branca)* (23%), Black Afro-Brazilian (*preta*) (10%), and East Asian-Brazilian (*amarela)* (2%). The median level of schooling in the cohort was 10 years (IQR: 8, 11), and the median monthly household income was 1.3 (IQR: 1.0, 2.2) times the Brazilian minimum wage in 2016.^30^ Overall, comorbidities during pregnancy were low, apart from gestational hypertension in 20% (*N* = 131) and anemia in 29% (*N* = 179) of participants. Notably, this prevalence of anemia during pregnancy is consistent with previous reports in Brazil.^31^ In the subset who underwent TORCH screening, small percentages of participants tested IgM-positive for Herpes simplex virus (10%), parvovirus B19 (2%), *Toxoplasma gondii* (1%), and cytomegalovirus (0.2%). Of the 694 participants included in the study, 305 (44%) had evidence of ZIKV infection and 145 (21%) had evidence of recent CHIKV infection. Excluding the 63 participants with IgM evidence of DENV infection, there remained a total of 326 participants who tested positive for either CHIKV or ZIKV infections (or both): 213 (31%) with ZIKV mono-infection, 55 (8%) with CHIKV mono-infection, and 58 (8%) with evidence of a sequential ZIKV/CHIKV infection (Table 2).

**Table 1:** Baseline characteristics of the MERG pregnancy cohort (*N* = 694) in Recife, Pernambuco, Brazil (2015–2017)

Characteristics		No. (% or IQR/±SD)	Missing values
Residency	Recife city	550 (79%)	–
	Outside Recife city	144 (21%)	
Age	Median years	26 (21,31)	–
Race/Ethnicity	Mixed-race Brazilian (*parda*)	448 (65%)	2
	White-Brazilian (*branca)*	163 (23%)	
	Black Afro-Brazilian (*preta*)	69 (10%)	
	East Asian-Brazilian (*amarela*)	12 (2%)	
Schooling	Median years	10 (8,11)	–
Highest education	Primary school (incl. equivalency program)	594 (86%)	7
	Secondary school (incl. equivalency program)	39 (6%)	
	Tertiary school incomplete	38 (6%)	
	Tertiary school complete	1 (2%)	
Inhabitants per household	Median number	3 (2, 4)	–
Monthly household income	Median income in BRL/month	1140 (877, 1915)	111
	Median income relative to minimum wage in 2016 (880 BRL/month)	1.30 (1.0, 2.2) × minimum wage	111
Comorbidities during pregnancy*	Anemia	179 (29%)	71
	Gestational hypertension	131 (20%)	23
	Diabetes	19 (3%)	3
	Hypothyroidism	5 (0.7%)	–
	Chronic kidney disease	2 (0.2%)	4
TORCH testing	Herpes simplex virus IgM	34 (10%)	360
	Parvovirus B19 IgM	7 (2%)	249
	*Toxoplasma gondii* IgM	4 (1%)	258
	Cytomegalovirus IgM	1 (0.2%)	210
Gestational trimester with reported rash	First	144 (19%)	106
	Second	22 (38%)	
	Third	248 (42%)	
Prior pregnancies	0	261 (38%)	–
	1	220 (32%)	
	2	109 (15%)	
	≥ 3	104 (15%)	
Previous adverse pregnancy outcomes†	Congenital anomalies	18 (5%)	303
	Stillbirth	17 (5%)	367
	Abortions (spontaneous or induced)	137 (38%)	332
	Mean no. of abortions among individuals with abortions ± SD	1.3 ± 0.72	–

BRL = Brazil release; IQR = interquartile range; MERG = Microcephaly Epidemic Research Group; SD = standard deviation.

* Diabetes was assessed before and during pregnancy; all other conditions were reported to have been diagnosed during pregnancy.

**Table 2:** Arbovirus diagnostic test results of the MERG pregnancy cohort (*N* = 694) in Recife, Pernambuco, Brazil (2015–2017)

	Testing methods	No. (%)
All ZIKV infections*†	ZIKV qRT-PCR, IgM, IgG3, and/or PRNT_50_	305 (44%)
All CHIKV infections*	CHIKV IgM	145 (21%)
ZIKV mono-infections†	ZIKV qRT-PCR, IgM, IgG3, and/or PRNT_50_	213 (31%)
CHIKV mono-infections	CHIKV IgM	55 (8%)
Sequential ZIKV/CHIKV infections†	(ZIKV qRT-PCR, IgM, IgG3, and/or PRNT_50_) *& *(CHIKV IgM)	58 (8%)

CHIKV = Chikungunya virus; MERG = Microcephaly Epidemic Research Group; ZIKV = Zika virus.

* The 305 ZIKV infections and 145 CHIKV infections include 63 cases with additional recent DENV infections who were excluded from further analyses.

† ZIKV test results interpreted using the MERG diagnostic algorithm.^22^

### Temporal and geographical investigation of CHIKV and ZIKV co-circulation.

**Figure 1.**
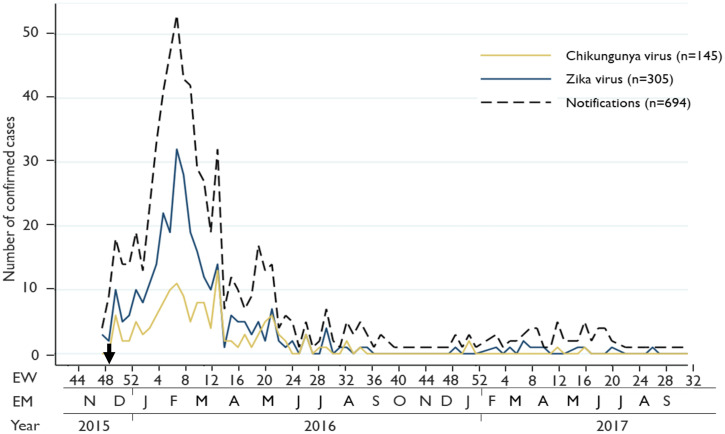
Epidemiological curve depicting all notified pregnant individuals that tested positive for ZIKV (blue) and CHIKV (yellow) and all pregnant individuals that were notified with rash (black dashes) in the cohort study in Recife, Pernambuco in Brazil (December 2015 to July 2017). CHIKV = Chikungunya virus; EM = epidemiological month; EW = epidemiological week; EY = epidemiological year; ZIKV = Zika virus. The black arrow indicates the beginning of the Cievs/PE surveillance system. This figure appears in color at www.ajtmh.org.

**Figure 2.**
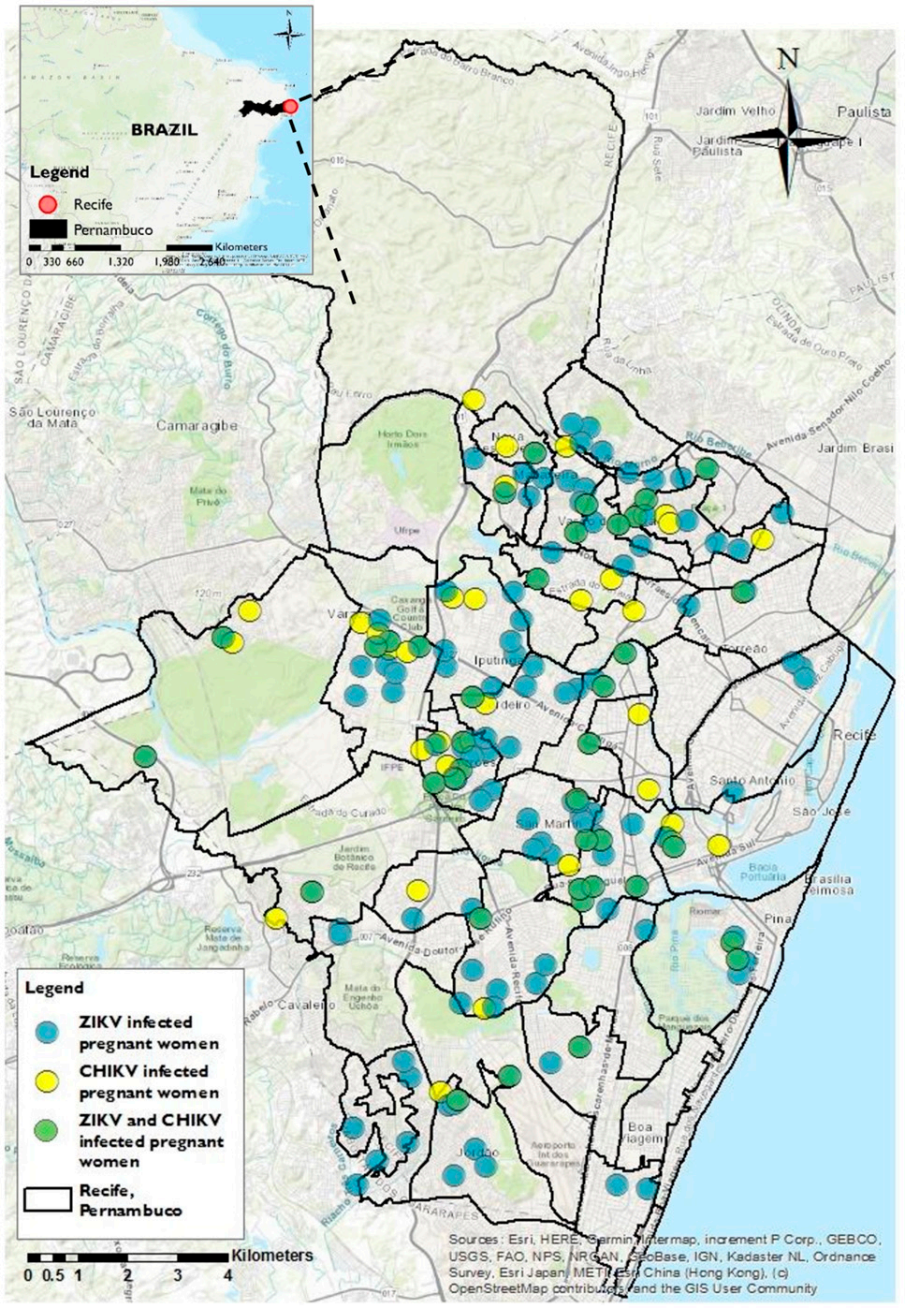
Map of all notified pregnant individuals that tested positive for ZIKV (blue) (*N* = 108) and CHIKV (yellow) (*N* = 34) and for ZIKV and CHIKV (green) (*N* = 38) in the cohort study in the city of Recife, Pernambuco in Brazil (December 2015 to July 2017). Recife is located in the East of Pernambuco, and Pernambuco is located in the northeast (small map at the top left). CHIKV = Chikungunya virus; ZIKV = Zika virus. This figure appears in color at www.ajtmh.org.

The epidemiological curve provides evidence of CHIKV and ZIKV co-circulation between December 2015 and June 2017 (Figure 1). The majority of CHIKV and ZIKV infections occurred between December 2015 and May 2016, during which a peak of up to 30 ZIKV cases and up to 15 CHIKV cases were detected among pregnant persons with rash per week. From June 2016 to August 2016, numbers of CHIKV and ZIKV infections decreased to fewer than five infections detected per week, followed by no incident infections between September and November 2016. Isolated CHIKV and ZIKV infections occurred in the cohort between December 2016 and June 2017. Of note, the expected seasonal pattern in northeast Brazil in the coastal areas, such as Recife, is moderate rains in “summer” (i.e., December to February) and strong rains in “winter” (i.e., April to July). Thus, while there is large growth of the mosquito population in summer, which leads to increased arbovirus infections, mosquito eggs are washed away in the winter rains, consistent with the temporal distribution of arbovirus infections detected in this study.^32^ Notably, the positive rates for CHIKV and ZIKV testing remained relatively consistent over the study period, suggesting the peaks of CHIKV and ZIKV cases depicted in the epidemiological curve reflect epidemic dynamics rather than changes in notification patterns (Supplemental Table 1). Of the 326 participants who tested positive for either CHIKV or ZIKV infections, 180 (55%) resided in Recife and had available residence data. The mapping indicated that these 180 pregnant individuals with CHIKV, ZIKV, and sequential ZIKV/CHIKV infections lived in highly overlapping regions of the city (Figure 2). Using a significance threshold of *P* = 0.001 for the Moran’s I index, there was insufficient evidence to reject the null hypothesis of zero spatial autocorrelation in the geographic distributions of maternal CHIKV (*P* = 0.039) or ZIKV (*P* = 0.003) infections.

### Clinical presentation of CHIKV and ZIKV infection.

After excluding the 63 participants who tested IgM-positive for DENV (i.e., 32 participants with evidence of ZIKV and 31 with evidence of CHIKV), clinical presentations were compared in 213 participants with ZIKV mono-infections, 55 with CHIKV mono-infections, and 58 with sequential ZIKV/CHIKV infections.

Among this group of pregnant persons with rash, individual clinical signs and symptoms were more frequently reported in CHIKV mono-infected participants and sequentially ZIKV/CHIKV infected than in ZIKV mono-infected participants (Table 3). This pattern was observed for nearly all symptoms (i.e., joint pain, headache, muscle pain, back ache, fatigue, joint swelling, nausea, and retro-orbital pain), apart from fever, photophobia, abdominal pain, and eye redness, which occurred with similar frequency in all three groups. Of note, participants who tested negative for all arboviruses presented with a similar clinical presentation to those with ZIKV mono-infections. After adjustment for potential confounding by maternal age, ZIKV mono-infected individuals had lower odds of presenting with arthralgia (aOR, 99% CI, *P* value: 0.33, 0.15–0.75, 0.001), arthritis (0.35, 0.14–0.85, 0.002), fatigue (0.40, 0.17–0.96, 0.007) and headache (0.44, 0.19–1.00, 0.01) than CHIKV mono-infected cases (Table 4). Further predictive modeling indicated no substantive improvements to model fit (i.e., differentiating CHIKV versus ZIKV mono-infections) when symptoms were combined in the model as compared with when signs and symptoms were included individually (Supplemental Table 2), likely owing to the high degree of correlation in the reporting of signs and symptoms (Supplemental Figure 1).

**Table 3:** Prevalence of signs and symptoms among Dengue virus (DENV)-IgM-negative pregnant persons with rash (*N* = 631) who tested negative for Zika (ZIKV), and Chikungunya (CHIKV) viruses (*N* = 305), positive for ZIKV mono-infection (*N* = 213), positive for CHIKV mono-infection (*N* = 55), and positive for sequential ZIKV/CHIKV infection (*N* = 58)

	Prevalence of signs and symptoms				
Signs and symptoms	Total (n_total_ = 631), n/N (%)	Negative for ZIKV, and CHIKV, (n_total_ = 305), n/N (%)	Positive for ZIKV, (n_total_ = 213), n/N (%)	Positive for CHIKV, (n_total_ = 55), n/N (%)	Positive for ZIKV/CHIKV (n_total_ = 58), n/N (%)
Fever	473/606 (78%)	216/287 (75%)	167/208 (80%)	44/54 (81%)	46/57 (81%)
Joint pain (arthralgia)	200/607 (33%)	79/289 (27%)	64/207 (31%)	31/54 (57%)	26/57 (46%)
Headache	170/606 (28%)	68/288 (24%)	55/208 (26%)	24/53 (45%)	23/57 (40%)
Muscle pain (myalgia)	163/607 (27%)	65/289 (22%)	54/207 (26%)	22/54 (41%)	22/57 (39%)
Back ache	129/605 (21%)	49/287 (17%)	43/208 (21%)	18/53 (34%)	19/57 (33%)
Fatigue	114/607 (19%)	48/288 (17%)	37/208 (18%)	19/54 (35%)	21/57 (37%)
Joint swelling (arthritis)	91/607 (15%)	30/289 (10%)	32/207 (15%)	19/54 (35%)	10/57 (18%)
Nausea	88/604 (15%)	39/286 (13%)	24/207 (12%)	11/54 (20%)	14/57 (25%)
Photophobia	60/603 (10%)	27/287 (9%)	19/207 (9%)	7/52 (13%)	7/57 (12%)
Retro-orbital pain	72/603 (12%)	37/288 (12%)	18/206 (9%)	11/52 (21%)	6/57 (11%)
Abdominal pain	46/605 (8%)	15/287 (5%)	18/207 (9%)	7/54 (13%)	6/57 (11%)
Eye redness	62/573 (11%)	31/257 (11%)	18/206 (9%)	6/53 (11%)	7/57 (12%)

CHIKV = Chikungunya virus; DENV = Dengue virus; ZIKV = Zika virus.

**Table 4:** The crude and adjusted odds ratio, among pregnant persons with rash, of presenting with a given signs or symptom comparing Zika (ZIKV) mono-infections or sequential ZIKV/Chikungunya (CHIKV) infections to CHIKV mono-infections

Signs and symptoms	Number of observations (*N* = 326)	Infection	Unadjusted		*P *value	Adjusted for maternal age		*P* value
			OR	(99% CI)		OR	(99% CI)	
Fever	319	CHIKV	1			1		
		ZIKV	0.92	0.34–2.53	*0.84*	0.90	0.33–2.48	*0.79*
		ZIKV/CHIKV	0.95	0.27–3.32	*0.92*	0.95	0.27–3.32	*0.91*
Joint pain (arthralgia)	318	CHIKV	1			1		
		ZIKV	0.33	0.15–0.74	0.0001	0.33	0.15–0.75	*0.001*
		ZIKV/CHIKV	0.62	0.23–1.67	0.22	0.62	0.23–1.67	*0.215*
Headache	318	CHIKV	1			1		
		ZIKV	0.43	0.19–0.98	*0.009*	0.44	0.19–1.0	*0.010*
		ZIKV/CHIKV	0.81	0.30–2.21	*0.60*	0.81	0.30–2.20	*0.60*
Muscle pain (myalgia)	318	CHIKV	1			1		
		ZIKV	0.51	0.23–1.17	*0.037*	0.52	0.23–1.18	*0.041*
		ZIKV/CHIKV	0.91	0.34–2.49	*0.82*	0.91	0.34–2.50	*0.82*
Back ache	318	CHIKV	1			1		
		ZIKV	0.51	0.21–1.21	*0.044*	0.53	0.22–1.26	*0.058*
		ZIKV/CHIKV	0.97	0.34–2.75	*0.94*	0.97	0.34–2.75	*0.93*
Fatigue	319	CHIKV	1			1		
		ZIKV	0.40	0.16–0.95	*0.006*	0.40	0.17–0.96	*0.007*
		ZIKV/CHIKV	1.07	0.39–2.98	*0.86*	1.07	0.39–2.98	*0.89*
Joint swelling (arthritis)	318	CHIKV	1			1		
		ZIKV	0.34	0.14–0.82	*0.002*	0.35	0.14–0.85	*0.002*
		ZIKV/CHIKV	0.39	0.12–1.25	*0.037*	0.39	0.12–1.25	*0.037*
Nausea	318	CHIKV	1			1		
		ZIKV	0.51	0.18–1.44	*0.096*	0.49	0.17–1.39	*0.077*
		ZIKV/CHIKV	1.27	0.39–4.13	*0.60*	1.27	0.39–4.15	*0.60*
Photophobia	316	CHIKV	1			1		
		ZIKV	0.64	0.19–2.19	*0.36*	0.63	0.19–2.14	*0.33*
		ZIKV/CHIKV	0.90	0.21–2.19	*0.85*	0.89	0.21–3.93	*0.85*
Retro-orbital pain	315	CHIKV	1			1		
		ZIKV	0.36	0.12–1.05	*0.014*	0.36	0.12–1.08	*0.017*
		ZIKV/CHIKV	0.44	0.11–1.80	*0.13*	0.44	0.11–1.80	*0.13*
Abdominal pain	318	CHIKV	1			1		
		ZIKV	0.63	0.19–2.17	*0.35*	0.60	0.17–2.04	*0.28*
		ZIKV/CHIKV	0.79	0.17–3.62	*0.69*	0.79	0.17–3.65	*0.69*
Eye redness	316	CHIKV	1			1		
		ZIKV	0.75	0.21–2.71	*0.56*	0.76	0.21–2.75	*0.58*
		ZIKV/CHIKV	1.10	0.24–5.04	*0.88*	1.10	0.24–5.04	*0.88*

CHIKV = chikungunya virus; ZIKV = Zika virus. For clinical features with an overall prevalence of < 5% in either group, logistic regressions were not performed due to low statistical power

Additionally after adjustment for maternal age, no signs and symptoms were found to be statistically significantly more or less common among sequentially ZIKV/CHIKV infected participants compared with CHIKV mono-infected participants (Tables 3 and 4), further challenging one’s ability to rule out ZIKV infections during pregnancy on the basis of symptoms.

## DISCUSSION

This study demonstrates geographic and temporal co-circulation of CHIKV and ZIKV in a cohort of pregnant persons presenting with rash and followed up between 2015 and 2017 in Recife, Pernambuco, Brazil. Overall, signs and symptoms—notably joint pain, joint swelling, fatigue and headache—were more frequently reported in participants with the evidence of maternal CHIKV infection than in those with the evidence of a maternal ZIKV mono-infection. However, there was a high degree of overlap in the clinical presentations that would make differential diagnosis between CHIKV and ZIKV infections based on symptoms alone unreliable in circumstances in which laboratory testing could not be performed in pregnant persons experiencing ZIKV-clinically compatible illness. Therefore, our findings reinforce the importance, in settings with arbovirus co-circulation, of laboratory diagnostic testing, especially in the context of pregnancy, to confirm infection type and to initiate appropriate follow-up.

Since 2015, when ZIKV was first discovered in Brazil, temporal and geographical CHIKV-ZIKV co-circulation has been frequently described throughout the Americas.^33^^–^^35^ Following introduction from Asia to the Caribbean island of Saint-Marteen, CHIKV emerged in Latin America and the Caribbean in 2013.^36^ By the beginning of 2014, CHIKV reached the Latin American mainland and quickly spread throughout the South American continent.^36^ Zika virus, meanwhile, was first identified in the northeast of Brazil in May 2015 before the epidemic began explosively expanding through the Americas.^27^^,^^28^ Evidence from the Brazilian Ministry of Health, confirms CHIKV-ZIKV co-circulation in Brazil and specifically in the Northeast of Brazil during the period of study (i.e., in 2016, 134 ZIKV and 415 CHIKV infections per 100,000 inhabitants registered in the northeast region; with 9 ZIKV and 249 CHIKV infections per 100,000 inhabitants registered in 2017).^37^^,^^38^

As a public health response to the microcephaly epidemic in 2015, the State Health Secretariat of Pernambuco made the notification of rashes and ZIKV infection during pregnancy compulsory beginning in December 2015.^22^ Therefore, the testing of this cohort study and the epidemic curve reflect the start of the awareness of the potential link between the microcephaly cases and the ZIKV outbreak, around 9 months after the actual beginning of the ZIKV outbreak in this region. Despite the timely response of the MERG to the public health emergency, the depicted peak of this study’s temporal epidemiological curve most likely does not reflect the true peak of the ZIKV outbreak but should be seen in the light of the new initiation of the surveillance system in December 2015. In line with the temporal findings of our study, the Brazilian Ministry of Health data indicates a decrease in CHIKV and ZIKV cases between 2016 and 2017.^37^^,^^38^

Of note, the temporal patterns of CHIKV and ZIKV infections in our study followed the expected patterns for coastal areas of northeast Brazil. In this cohort, we observed a high incidence of infections during the December to April from 2015 to 2016 and decreased incidence in June to November of 2016.^32^ This seasonal pattern for arbovirus infections in coastal areas of northeast Brazil has been described in two additional studies: a ZIKV study from 2015 to 2016 on a population sample (*N* = 260) in the city of Paulista in the Recife Metropolitan Region, geographically adjacent to our study, and a DENV time series analysis of surveillance data in the two Brazilian cities, Recife and Goiânia, between 2001 and 2014.^39^^,^^40^

To aid the differential diagnosis of CHIKV and ZIKV infections in a setting with co-circulation, an understanding of the relative frequency of clinical signs and symptoms of CHIKV and ZIKV in comparison to each other is important. Clinical symptom frequencies for CHIKV and ZIKV infections have commonly been reported in isolation from each other.^41^^–^^45^ Compared with our study, other studies have described higher symptom frequencies especially for joint pain, joint swelling, headache, myalgia, and retro-orbital pain in ZIKV mono-infections for both the pregnant and the general population.^20^^,^^41^^,^^43^^,^^45^ However, of these studies, two reported fever frequencies of around 35%, in contrast to the study of Duffy and others and our study, which report about double the fever frequency among ZIKV mono-infected cases.^41^^,^^43^^,^^45^ The symptom frequencies of CHIKV mono-infections observed in this study are largely supported by the findings of Bagno and others, apart from slightly increased frequencies of arthralgia and myalgia in comparison to our investigation and only a 40% symptom frequency of rash compared with the 100% in our study, as by design the participants were recruited into our cohort study on the basis of presenting with rash.^46^

Although our results suggest that the frequency of some of the clinical features of ZIKV mono-infections differ from that of CHIKV mono-infections, there remains substantial overlap in the presentations of sequential ZIKV/CHIKV infections and CHIKV mono-infections. To our knowledge only three studies have reported the clinical presentation of ZIKV mono-infections compared with that of CHIKV mono-infections.^35^^,^^47^^,^^48^ One review study, using data from different geographic sources, reported similar clinical presentation of the CHIKV, DENV, and ZIKV infections; however, the presented frequencies of clinical symptoms caused by ZIKV infections were based on a study population of 31 ZIKV-infected cases and on CHIKV and DENV in study populations of unreported sizes.^47^ Furthermore, this study did not describe how the comparison between the frequencies of clinical symptoms of ZIKV infections with that of CHIKV and DENV infections was statistically analyzed.^41^^,^^47^ A second study based in Brazil describes a more severe rash and conjunctival hyperemia for ZIKV cases compared with DENV and CHIKV cases, but did not describe their sample size or their methods used to assess signs and symptoms, diagnose cases or statistically compare frequency of signs and symptoms between ZIKV, CHIKV, and DENV infections.^48^ A study by Waggoner and others on 346 patients with suspected arboviral illness in Nicaragua reported no significant difference in symptom frequencies between 37 ZIKV cases and 103 CHIKV cases, with the exception of rash, which was reported in 91% of ZIKV cases but only 56% of CHIKV cases.^35^

Compared with other studies reporting on co-circulation of CHIKV and ZIKV, our study has benefited from a large cohort population with great detail on individual characteristics and of reported signs and symptoms.^35^^,^^47^^,^^48^ Nevertheless, our study had limitations. First, selection biases may have been introduced into the study as recruitment to the study was conditional on presenting with rash during pregnancy. Whereas rash has been reported to occur in approximately 95% of symptomatic ZIKV infected cases, it has been reported in only 56% of symptomatic CHIKV-infected cases in previous studies.^35^^,^^41^^,^^43^^,^^45^ In addition to limiting our understanding of the relative incidence of ZIKV and CHIKV infections in the general pregnant population, this prerequisite for notification limits the generalizability of our estimates of symptom frequency for chikungunya disease cases during pregnancy to the subset of infections presenting with rash. Second, information bias may have been introduced during the assessment of signs and symptoms of CHIKV and ZIKV infections, as many symptoms were self-reported and as all study participants were pregnant, any signs and symptoms were potentially more likely to be reported, as the pregnant population may have a heightened health-related awareness and lower threshold for reporting symptoms compared with the general population, particularly during this ZIKV outbreak. Nevertheless, as pathogen testing was performed blinded to clinical presentation, any misclassification of symptoms would have been non-differential related to the infection type and therefore expected to bias the estimates toward the null. Third, misclassification may have also arisen in the arbovirus testing owing to limitations in the sensitivity the laboratory tests given the timing of sample collection. Of note, the clinical presentations of the arbovirus test-negative group are very similar to the ZIKV-mono-infection group, which likely reflects some degree of false negative results. Ximenes and others describe that among pregnant individuals in this cohort who tested ZIKV qRT-PCR-positive, 58% were not observed to become IgM or PRNT_50_ positive upon subsequent testing.^22^ This risk of misclassification, however, was mitigated by conducting diagnostic tests for ZIKV infections using serially collected samples.^22^ The lack of qRT-PCR testing for CHIKV infections may have led to the missing of some CHIKV infections within the cohort as well as the misclassifications of rare ZIKV/CHIKV coinfections as ZIKV mono-infections.^49^ Also of note, given that positive qRT-PCR ZIKV results were detected up to 78 days after rash onset, we were unable to reliably discern the timing of CHIKV and ZIKV infections in relation to one another.^22^ Fourth, the clinical presentation assessed in this study may be specific to pregnancy, as immunological alterations during gestation may lead to altered clinical presentations.^50^^–^^52^ However, studies have described a very similar clinical presentation of CHIKV and ZIKV infections in pregnancy in comparison to that of the general population, apart from fever which has been described to be a less prevalent symptom of ZIKV infection in the pregnant population compared with in the nonpregnant population.^41^^,^^53^^–^^56^ Fifth, DENV neutralization testing was not performed in the diagnostic workup of this study due to the hyperendemicity of DENV in the study setting. As previously reported, approximately 95% of a subset of samples in the MERG Pregnancy Cohort tested positive for anti-DENV IgG,^22^ and > 85% of pregnant persons in a case-control study conducted in Recife during a similar time period were found to have neutralizing antibodies to DENV.^57^ While the extent to which prior DENV exposure may influence the clinical presentation of ZIKV remains uncertain, this may reduce the generalizability of our findings to flavivirus naïve populations.

In conclusion, our findings provide evidence of CHIKV and ZIKV co-circulation and underscore that the arboviruses’ clinical presentations overlap to such an extent that clinical symptoms alone are insufficient for reliable differential diagnosis. ZIKV infections cannot be ruled out in a patient with typical CHIKV symptoms (e.g., arthritis) as this may be a result of recent antecedent infection or coinfection. Thus, these data provide evidence that laboratory diagnostics continue to be an essential adjunct for reliably differentiating between maternal CHIKV and ZIKV infections in the event of CHIKV-ZIKV co-circulation. In addition to efforts for enhancing diagnostic and surveillance capacity, integrated arbovirus preventive strategies that strengthen vector control, improve housing and sanitation, and facilitate the use of personal protective measures should remain a priority.

## Supplemental Material


Supplemental materials
