# Supplementary material for: Co-circulation of Chikungunya Virus during the 2015–2017 Zika Virus Outbreak in Pernambuco, Brazil: An Analysis of the Microcephaly Epidemic Research Group Pregnancy Cohort
Source: Am J Trop Med Hyg. 2022 Apr 11;106(6):1711–20. doi: 10.4269/ajtmh.21-0449 (PMC9209936; doi:10.4269/ajtmh.21-0449)
Supplement: Supplementary file 1 [file tpmd210449.SD1.pdf]

Supplemental Material 1. Summary of the Microcephaly Epidemic Research Group diagnostic algorithm from Ximenes, et al. (2019) Zika virus infection in pregnancy: Establishing a case definition for clinical research on pregnant women with rash in an active transmission setting. PLoS Negl Trop Dis. 2019 Oct 7;13(10):e0007763.

"Reflecting the varying degrees of confidence in the specific laboratory assays, the timing of testing in relation to the dates of the rash and the pregnancy, and the availability of confirmatory test results, the expert panel developed and applied a set of definitional criteria to the dataset. Cases were defined as having robust evidence of maternal ZIKV infection if they had a positive nucleic acid amplification test, seroconversion, or at least two positive serologic tests in pregnancy of if they had one positive serologic tests (i.e., IgM or IgG3) in pregnancy paired with a non-negative PRNT50 within six months post-pregnancy. Cases were defined as having moderate evidence of maternal ZIKV infection if they had only one positive serologic test (i.e., IgM or IgG3) in pregnancy, an indication of seroconversion by PRNT50 during pregnancy (i.e., either a PRNT50 titer ≥1000 in pregnancy paired with a rise within 2 months post-pregnancy or 4-fold rise in PRNT50 titer from pregnancy to within 2 months post-pregnancy), or an equivocal PRNT50 test result in pregnancy paired with a positive PRNT50 within three months post-pregnancy. Cases were defined as having limited evidence of ZIKV infection but with uncertain timing relative to the pregnancy if they had a positive PRNT50 in pregnancy or within 6 months post-pregnancy or an indication of PRNT50 seroconversion during the 2 to 3 months post-pregnancy. Cases were defined as having limited evidence of a flavivirus before or during pregnancy if they had a PRNT50 titer between 20 and 100 or a non-negative result (i.e., unspecified titer ≥20) in pregnancy or within 1 month post-pregnancy. Finally, cases were considered to have evidence against ZIKV infection in pregnancy if all tests performed in pregnancy were negative."

Supplemental Table S1. Proportion of notified pregnant persons with rash that tested positive for ZIKV and CHIKV per two weeks from December 2015 to July 2017.

Weeks since 1 December 2015	No. of ZIKV positive	No. of CHIKV positive	No. of all persons notified	Proportion that tested positive for ZIKV	95% CI	Proportion that tested positive for CHIKV	95% CI
**1-2**	5	0	13	0.38	0.14- 0.68	0	-
**3-4**	15	8	32	0.47	0.29 - 0.65	0.25	0.12- 0.43
**5-6**	16	7	33	0.48	0.31 - 0.66	0.21	0.089-0.39
**7-8**	19	7	36	0.53	0.35 - 0.70	0.19	0.082-0.36
**9-10**	36	14	74	0.49	0.37 - 0.61	0.19	0.11-0.30
**11-12**	51	21	100	0.51	0.41 - 0.61	0.21	0.13-0.30
**13-14**	47	14	85	0.55	0.44 - 0.66	0.17	0.093 -0.26
**15-16**	28	16	56	0.50	0.36 - 0.64	0.29	0.17- 0.42
**17-18**	24	17	51	0.47	0.33- 0.62	0.33	0.21- 0.48
**19-20**	7	4	19	0.37	0.16 - 0.62	0.2	0.061-0.46
**21-22**	10	4	17	0.59	0.33 - 0.82	0.24	0.068-0.50
**23-24**	8	4	26	0.31	0.14 - 0.52	0.15	0.044-0.35
**25-26**	9	11	27	0.33	0.17 - 0.54	0.41	0.22- 0.61
**27-28**	3	5	10	0.30	0.067-0.65	0.5	0.19-0.81
**29-30**	2	0	6	0.33	0.043-0.78	0	-
**31-32**	3	3	6	0.50	0.12 - 0.88	0.5	0.12 -0.88
**33-34**	4	2	9	0.44	0.14-0.78	0.22	0.028-0.60
**35-36**	1	0	3	0.33	0.0084-0.91	0	-
**37-38**	1	2	8	0.13	0.0032-0.53	0.25	0.032-0.65
**39-40**	2	1	8	0.25	0.032-0.65	0.13	0.0032-0.53
**41-42**	0	0	4	0	-	0	-
**43-44**	0	0	3	0	-	0	-
**45-46**	0	0	1	0	-	0	-
**47-48**	0	0	1	0	-	0	-
**49-50**	0	0	1	0	-	0	-
**51-52**	0	0	1	0	-	0	-
**53-54**	1	0	4	0.25	0.0063-0.81	0	-
**55-56**	0	2	4	0	-	0.5	0.068-0.93
**57-58**	1	0	4	0.25	0.0063-0.81	0	-
**59-60**	1	0	4	0.25	0.0063-0.81	0	-
**61-62**	3	0	7	0.43	0.099-0.82	0	-
**63-64**	2	0	5	0.40	0.053-0.86	0	-
**65-66**	0	1	6	0	-	0.17	0.053-0.85
**67-68**	0	0	2	0	-	0	-

**69-70**	1	1	3	0.33	0.0042-0.64	0.33	0.0042-0.64
**71-72**	1	0	3	0.33	0.0042-0.64	0	-
**73-74**	0	0	3	0	-	0	-
**75-76**	0	0	8	0	-	0	-
**77-78**	1	0	2	0.50	0.013-0.99	0	-
**79-80**	0	0	2	0	-	0	-
**81-82**	0	0	1	0	-	0	-
**83-84**	1	0	2	0.50	0.013-0.99	0	-
**85-86**	0	0	1	0	-	0	-

Supplemental Table S2. Odds ratio of being ZIKV mono-infected compared to CHIKV mono-infected by symptom presentation among pregnant persons with rash, comparing models combining symptoms versus a model based on joint pain alone.

Symptoms included in the model	Variable	Odds ratio of being ZIKV infected vs. CHIKV infected	95% CI	Comparison with M1 p-value, Likelihood Ratio Test
M1. Joint pain	Joint pain	0.33	(0.18 - 0.61)	--
M2. Joint pain + joint swelling	Joint pain	0.43	(0.21 - 0.88)	*0.15*
	Joint swelling	0.56	(0.25 - 1.23)	
M3. Joint pain + fatigue	Joint pain	0.40	(0.20 - 0.79)	*0.28*
	Fatigue	0.65	(0.31 - 1.41)	
M4. Joint pain + headache	Joint pain	0.36	(0.17 - 0.79)	*0.73*
	Headache	0.87	(0.39 - 1.92)	
M5. Joint pain + joint swelling + fatigue +headache	Joint pain	0.46	(0.20 - 1.06)	*0.47*
	Joint swelling	0.61	(0.26 - 1.41)	
	Fatigue	0.74	(0.33 - 1.69)	
	Headache	1.05	(0.45 - 2.44)	

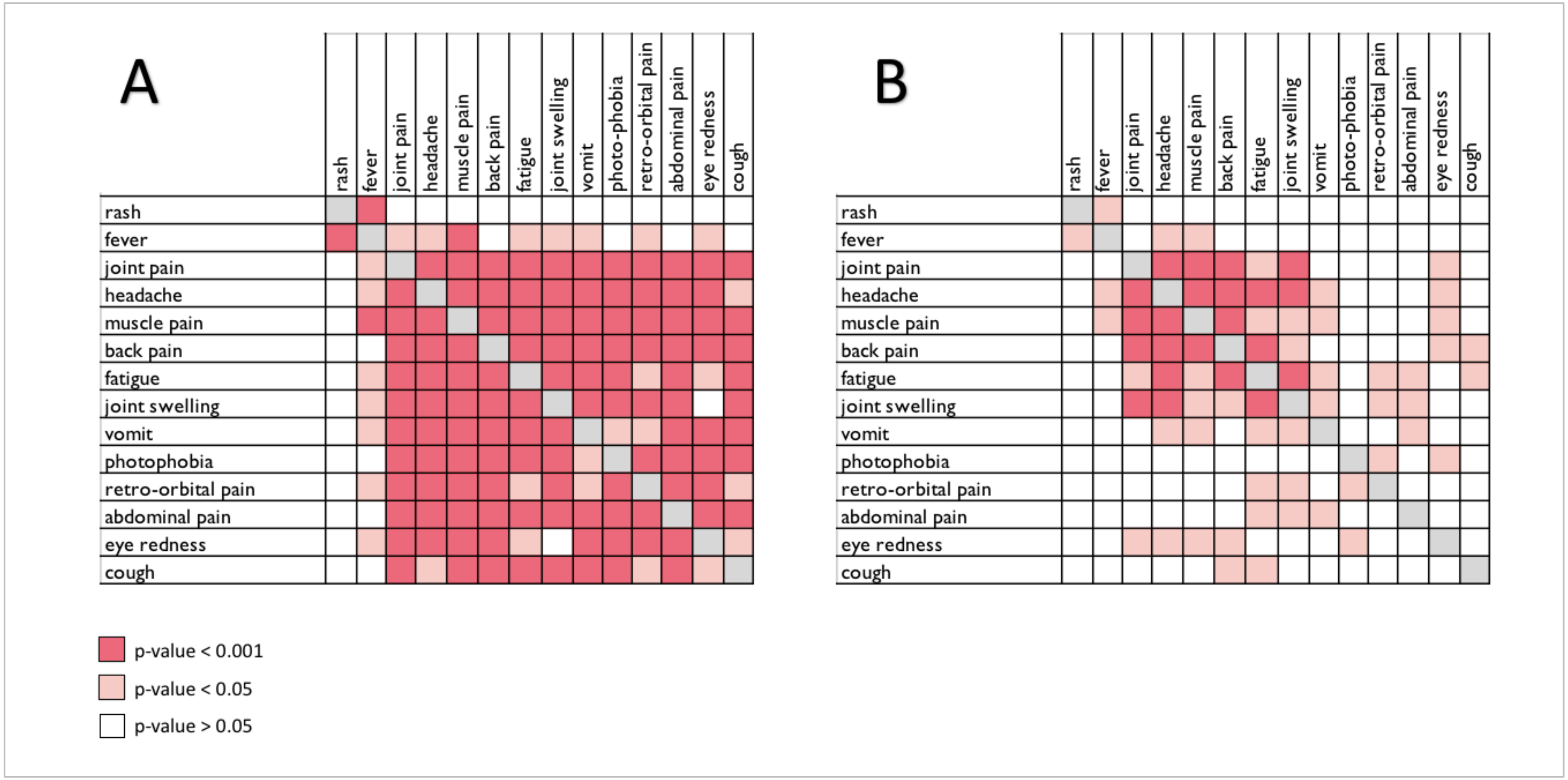

Supplemental Figure S1. Strength of pairwise associations between symptoms. In **A** association of symptom presentation is presented only for Zika virus (ZIKV) cases and in **B** association of symptom presentation is presented only for CHIKV cases. Association was tested using chi-squared test. Dark red depicted a p-value of <0.001, pink depicted a p-value of <0.05 and white depic
